# Effectiveness comparisons of catgut implantation at acupoint for obese type 2 diabetes: A protocol for systematic review and meta analysis

**DOI:** 10.1097/MD.0000000000021316

**Published:** 2020-07-24

**Authors:** Chunli Piao, Qi Zhang, Huiyan Fu, Li Wang, Cheng Tang

**Affiliations:** a Shenzhen Hospital (Futian), Guangzhou University of Chinese Medicine, Shenzhen, Guangdong Province; b Changchun University of Chinese Medicine, Changchun, Jilin Province; c The Third Affiliated Hospital of Xuzhou Medical University, Xuzhou, Jiangsu Province, China.

**Keywords:** Acupoint catgut embedding, obese type 2 diabetes, protocol, systematic review

## Abstract

**Background:**

With the change of people's life style, many more people are suffering from obese type 2 diabetes mellitus (T2DM). Acupoint catgut embedding is one of the acupuncture treatment principles in traditional Chinese medicine, which is widely used in the treatment of obese T2DM. However, there is no systematic review of the therapeutic effect of acupoint catgut embedding on obesity T2DM. Therefore, this article aims at the meta-analysis of acupoint catgut embedding in the treatment of obese T2DM, to clarify its curative effect.

**Methods:**

A structured and systemic literature search was conducted in the following databases up to December 1, 2019: PubMed, Cochrane Central Register of Controlled Trials (CENTRAL), Web of Science, EMBASE, CNKI, Wanfang Database. We will use the Review Manager 5.3 software provided by Cochrane collaborative network for statistical analysis. Then we assessed the quality and risk of the included studies and observed the outcome measures.

**Results:**

This meta-analysis will further determine the beneficial efficacy of acupoint catgut embedding on obesity T2DM.

**Conclusion:**

The purpose of this meta-analysis is to explore the effect of acupoint catgut embedding intervention on obese T2DM patients, and provide more options for clinicians and patients to treat obese T2DM.

**Ethics and dissemination:**

This systemic review will evaluate the efficacy and safety of acupoint catgut embedding in the treatment of obesity T2DM. Since all the data included are published, the systematic review does not need ethical approval.

**Registration number:**

CRD42020160801.

## 1 Introduction

With the changes in diet and lifestyle, obesity has become an alarming health crisis of the 21st century.^[1]^ In general, the most important comorbidity of obesity is type 2 diabetes mellitus (T2DM), as it plays a central role in the development of other comorbidities and further aggravates the metabolic syndrome.^[2]^ Obesity is a major risk factor for the development of T2DM, as the associated chronic, low-grade, sterile inflammation contributes to both insulin resistance and β-cell failure.^[3]^ In the United Kingdom (UK), of the 3.2 million people with diabetes, an estimated 80% to 85% are overweight or obese, in whom weight gain could be potentially detrimental.^[4]^ More and more evidences suggest that weight loss has clinical significance in improving blood glucose control, lipid status, renal function, blood pressure, and quality of life for adults with obesity T2DM.^[5–7]^ Currently, metabolic surgery is the most effective way to treat obesity T2DM.^[8,9]^ However, there are some disadvantages in metabolic surgery, such as infection, dyspepsia, and high cost.^[10]^ Empirical data also suggest that mortality rates with metabolic operations are typically 0.1% to 0.5%.^[11]^ Thus, further treatment strategies, including integrated Chinese medicine, metabolic surgery, and routine pharmacology, are still urgently needed for obese T2DM.

Acupoint catgut embedding is a treatment method originated from traditional acupuncture. For instance, the book “Magic Pivot, End and Beginning” has mentioned “those who have been ill for a long time, the evil spirit goes deep, and those who have been stabbing this disease will stay for a long time.”^[12]^ Catgut embedding acupuncture is an updated and improved form of classic manual acupuncture with the advantages of lowering expense and time, as well as longer lasting stimulation without additional biological effect in comparison with manual acupuncture.^[13]^ In recent years, Catgut embedding at acupoints is becoming much more popular in the treatment of obesity and diabetes. However, there is no systematic review of the therapeutic effect of acupoint catgut embedding on obesity T2DM. As we have seen, this is the first meta-analysis of RCT to explore the effect of acupoint catgut embedding intervention on obese T2DM patients.

## 2 Methods

This protocol which has been reported is based on the Preferred Reporting Items for Systematic Reviews and Meta-Analyses Protocols (PRISMA-P) guideline^[14]^ and the corresponding checklist used. This systematic review protocol was registered on the PROSPERO international registry of systematic reviews (ID: CRD42020160801).

### 2.1 Inclusion criteria

#### 2.1.1 Types of participants

Participants with standards of body mass index (BMI) ≥24 kg/m^2^ which are diagnosed with T2DM will be included. The diagnosis of T2DM is in accordance with who's diagnostic criteria for diabetes in 1999.^[15]^ Meanwhile, the participants who are included have no limitation of age, sex, region, citizenship, and nationality. Cases related to serious diseases, pregnancy, and drug-induced obesity are excluded.

#### 2.1.2 Types of interventions

Observation group: They received intervention using acupoint catgut embedding for a minimum duration of 4 weeks. The intervention method can be acupoint catgut embedding alone or acupoint catgut embedding combined with other therapies.

Control group: Patients in the control group were treated with sham acupuncture, placebo, or hypoglycemic drugs. However, acupuncture combined with drugs will be excluded.

#### 2.1.3 Types of outcome measures

The primary outcome measures were fasting blood glucose, 2 hours postprandial blood glucose (2hG), glycosylated hemoglobin, and BMI. The second observation index mainly includes triglyceride (TG), cholesterol (TC), insulin secretion (INS), waist circumference, hip circumference, and so on. The adverse events as safety outcomes will be reported.

#### 2.1.4 Types of studies

Randomized controlled clinical trials and quasi-randomized controlled trials will be included.

### 2.2 Data source

A structured and systemic literature search was conducted in the following databases up to December 1, 2019: PubMed, Cochrane Central Register of Controlled Trials (CENTRAL), Web of Science, EMBASE, CNKI, Wanfang Database. The language of the selected literature includes Chinese and English. The search terms include: obese T2DM; obesity and T2DM; acupoint catgut embedding; catgut embedding; catgut implantation.

**Figure 1:**
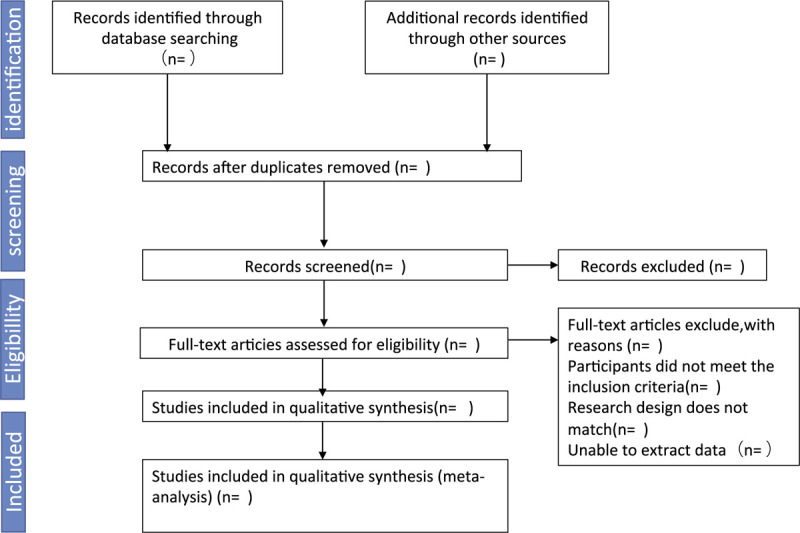
PRISMA flow diagram of study inclusion and exclusion.

Searching other resources: The manual search mainly is used for searching relevant studies, such as “Chinese Journal of Endocrinology and Metabolism” before the database creation. Details of the selection process were shown in the flow chart and the screening process is summarized in a flow diagram (Fig. 1).

### 2.3 Selection of studies

At first, according to the inclusion and exclusion criteria, two researchers (WL and TC) independently selected the literature after reading the title and abstract. Secondly, by reading the full text, we exclude uncontrolled research, nonrandomized, inconsistent evaluation criteria and similar data. If any differences occur during the screening study, the third author (P-CL) would be intervened.

### 2.4 Data extraction and management

Two researchers (WL and TC) used a predesigned data extraction table to extract the data of the final included study, including author, year, sample size, course of treatment, intervention measures, outcome indicators, adverse reactions, among others. The process of study selection will be performed by using the methods according to the PRISMA guidelines, presenting in the flow diagram.

### 2.5 Statistical analysis

We will use the Review Manager 5.3 software provided by Cochrane collaborative network for statistical analysis. For continuous variables, mean and SD of each study were obtained and pooled as mean difference (MD) or standardized mean differences (SMD) with a 95% confidence interval (CI).^[16]^ Statistical heterogeneity analysis was carried out for the Clinical RCTs included. Cochrane *I*^2^ test was used. When *I*^2^ was <50% or *P* > .05, it indicated that there was no statistical heterogeneity between the studies, the fixed-effect model was selected to combine the effect amount; otherwise, the random effect model was adopted.

### 2.6 Methodological quality of assessment

The literature quality of this study was evaluated by the bias risk table proposed by Cochrane collaborative network. The risk table includes 6 items: random sequence generation mode, whether to use allocation concealment, whether to blind the subjects and intervention providers, whether to blind the results evaluators, whether the results data are complete, whether to select the results report and other bias sources. The criteria used to assess the risk of bias are “low risk,” “high risk,” and “unclear.” In this process, 2 evaluators independently evaluate the methodological quality. In case of disagreement, the third author would be intervened.

### 2.7 Assessment of heterogeneity

If there was no significant heterogeneity (*I*^2^ < 50%) between a group of studies, the fixed-effect model was used for evaluation. If there is significant heterogeneity (*I*^2^ > 50%) between a group of studies, the random-effect model is used for evaluation, and then sensitivity analysis or subgroup analysis is conducted as required to explain the heterogeneity.

### 2.8 Assessment of publication bias

If there are >10 trials in accordance with the study, we can use Rev man5.3 software to draw and analyze the funnel chart, and use the funnel chart to evaluate the potential publication bias.

### 2.9 Grading the quality of evidence

It is recommended to use the Grading of Recommendations Assessment, Development and Evaluation (GRADE)^[17]^ to analyze the quality level of evidence.

## 3 Discussion

In recent years, with the change of people's lifestyle and the increase of work pressure, the incidence of diabetes is increasing. In the occurrence and development of diabetes, there will be many complications, such as obesity. Obesity is not only its complication, but also its inducing factor. Insulin resistance is the common basis of obesity and T2DM.^[18]^ The rise in prevalence of obesity coincides with the prevalence of T2DM. It has been estimated that by the year 2040, a staggering 642 million people will suffer from this disease worldwide.^[19]^ In this connection, many doctors have explored many methods for the treatment of this disease, which has also been widely applied in clinical practice, such as oral medication, or surgical treatment. However, there are some deficiencies in these treatments. When the drugs are taken orally, there will be effects on the liver and kidney function of the human body, or when the drugs are stopped, the disease will be repeated. The risk of infection also exists in surgical treatment.^[20]^

Traditional Chinese medicine believes that the formation of obese diabetes is mainly due to the irregular diet, excessive diet, fat, sweet, and thick taste, whereas the internal factors of the formation of the disease are the disorder of spleen and stomach and the accumulation of phlegm and dampness.

It is recorded in the book of Treatise On strange diseases that “the fat make people hot inside, and the sweet make people full inside.” Obese people tend to have copious phlegm. Therefore, this kind of disease is mostly based on deficiency in origin and excess in superficiality.

Traditional Chinese medicine research shows that acupuncture and moxibustion can play an auxiliary role in controlling blood sugar and reducing weight.^[21]^ Acupoint catgut embedding therapy is an improved application of acupuncture and moxibustion. It can produce continuous physical stimulation on acupoints to achieve the therapeutic purpose. Meanwhile, Catgut Embedding at acupoints is also widely used in clinical practice.

However, there is no systematic meta-analysis of the therapeutic effect of Catgut Embedding at acupoints on obese T2DM. Therefore, we plan to make a systematic review of acupoint catgut embedding therapy for obese T2DM, so as to provide high-quality evidence for the effectiveness and safety of acupoint catgut embedding therapy for obese T2DM.

## 4 Limitations

However, we cannot avoid the following problems when we collect literature: the quality of literature is not high enough; the literature only includes English and Chinese, which is not comprehensive enough; the sample size of the included literature is not large enough.

## Author contributions

**Conceptualization:** Chunli Piao, Qi Zhang

**Data curation:** Cheng Tang, Li Wang

**Data synthesis:** Cheng Tang, Li Wang

**Methodology:** Chunli Piao, Qi Zhang

**Software:** Chunli Piao, Qi Zhang

**Writing draft:** Chunli Piao, Qi Zhang, Huiyan Fu

## References

[1] Li B Cheng Y Yu S Human umbilical cord-derived mesenchymal stem cell therapy ameliorates nonalcoholic fatty liver disease in obese type 2 diabetic mice. Stem Cells Int 2019;8628027. 31781248 10.1155/2019/8628027 PMC6875176

[2] Madadi F Jawad R Mousati I Remission of type 2 diabetes and sleeve gastrectomy in morbid obesity: a comparative systematic review and meta-analysis. Obes Surg 2019;29:4066–76. 31655953 10.1007/s11695-019-04199-3

[3] He W Yuan T Maedler K Macrophage-associated pro-inflammatory state in human islets from obese individuals. Nutrition Diabetes 2019;9:36. 31787760 10.1038/s41387-019-0103-z PMC6885511

[4] Diabetes U State of the Nation Challenhes for 2015 and beyond. London 2014.

[5] Cai X Qiu SH Yin H Pedometer intervention and weight loss in overweight and obese adults with type 2 diabetes: a meta-analysis. Diabet Med 2016;33:1035–44. 26926674 10.1111/dme.13104 PMC5071725

[6] Cabre N Luciano-Mateo F Baiges-Gaya G Plasma metabolic alterations in patients with severe obesity and non-alcoholic steatohepatitis. Aliment Pharmacol Ther 2019;51:374–87. 31825539 10.1111/apt.15606

[7] Giagulli VA Castellana M Murro I The role of diet and weight loss in improving secondary hypogonadism in men with obesity with or without type 2 diabetes mellitus. Nutrients 2019;11:12. 10.3390/nu11122975 PMC6950423 31817436

[8] Perez-Pevida B Escalada J Miras AD Mechanisms underlying type 2 diabetes remission after metabolic surgery. Front Endocrinol 2019;10:641. 10.3389/fendo.2019.00641 PMC6761227 31608010

[9] Yan G Wang J Zhang J Long-term outcomes of macrovascular diseases and metabolic indicators of bariatric surgery for severe obesity type 2 diabetes patients with a meta-analysis. PloS One 2019;14:e0224828. 31794559 10.1371/journal.pone.0224828 PMC6890174

[10] Catoi AF Parvu A Muresan A Metabolic mechanisms in obesity and type 2 diabetes: insights from bariatric/metabolic surgery. Obesity facts 2015;8:350–63. 26584027 10.1159/000441259 PMC5644813

[11] American Diabetes Association. 8. Obesity management for the treatment of type 2 diabetes: Standards of Medical Care in Diabetes—2020. Diabetes Care 2020;43: suppl 1: S89–97. 31862751 10.2337/dc20-S008

[12] Wu X-Y Chen G-Z Li Y-T The key issues and corresponding strategy of acupoint medicated catgut embedding therapy. Acupuncture in China 2019;39:81–5. 10.13703/j.0255-2930.2019.01.019 30672262

[13] Thingholm LB Ruhlemann MC Koch M Obese individuals with and without type 2 diabetes show different gut microbial functional capacity and composition. Cell host & microbe 2019;26:252–64. e10. 31399369 10.1016/j.chom.2019.07.004 PMC7720933

[14] Moher D Shamseer L Clarke M Preferred reporting items for systematic review and meta-analysis protocols (PRISMA-P) 2015 statement. Syst Rev 2015;4:1 doi: 10.1186/2046-4053-4-1. 25554246 10.1186/2046-4053-4-1 PMC4320440

[15] Gabir MM Hanson RL Dabelea D The 1997 American Diabetes Association and 1999 World Health Organization criteria for hypergiycemia in the diagnosis and prediction of diabetes. Diabetes Care 2000;23:1108–12. 10937506 10.2337/diacare.23.8.1108

[16] Zhang Yang L Shergis J Chinese herbal medicine for diabetic kidney disease: a systematic review and meta-analysis of randomised placebo-controlled trials. BMJ Open 2019;9:e025653. 10.1136/bmjopen-2018-025653 PMC6501976 31048437

[17] Langer G Meerpohl JJ Perleth M GRADE guidelines: 12. Developing summary of findings tables—dichotomous outcomes. Z Evid Fortbild Qual Gesundhwes 2013;107:646–64. 24315336 10.1016/j.zefq.2013.10.034

[18] Akshintala D Chugh R Amer F Feingold KR Anawalt B Boyce A Chrousos G Dungan K Grossman A MDText.com, Inc, Nonalcoholic Fatty Liver Disease: The Overlooked Complication of Type 2 Diabetes. Endotext. South Dartmouth (MA): 2000. 31310460

[19] International Diabetes Federation (IDF). IDF Diabetes Atlas. 7th ed. Brussels, Belgium: IDF; 2015. doi:10.1289/image.ehp.v119.i03.

[20] Jespersen MJ Knop FK Christensen M GLP-1 agonists for type 2 diabetes: pharmacokinetic and toxicological considerations. Expert opinion on drug metabolism & toxicology 2013;9:17–29. 23094590 10.1517/17425255.2013.731394

[21] Dai C Chen G Yang J Effect of acupuncture combined with sitagliptin on obese patients with type 2 diabetes and its effect on adiponectin,adropin and irisin. Liaoning Journal of traditional Chinese Medicine 2019;46:2172–5.

